# Music Perception Testing Reveals Advantages and Continued Challenges for Children Using Bilateral Cochlear Implants

**DOI:** 10.3389/fpsyg.2019.03015

**Published:** 2020-01-21

**Authors:** Morrison M. Steel, Melissa J. Polonenko, Sara Giannantonio, Talar Hopyan, Blake C. Papsin, Karen A. Gordon

**Affiliations:** ^1^Archie’s Cochlear Implant Laboratory, The Hospital for Sick Children, Toronto, ON, Canada; ^2^Department of Otolaryngology-Head and Neck Surgery, University of Toronto, Toronto, ON, Canada

**Keywords:** deafness, cochlear implant, electrical stimulation, Montreal Battery of Evaluation of Amusia (MBEA), bilateral, memory

## Abstract

A modified version of the child’s *Montreal Battery of Evaluation of Amusia* (cMBEA) was used to assess music perception in children using bilateral cochlear implants. Our overall aim was to promote better performance by children with CIs on the cMBEA by modifying the complement of instruments used in the test and adding pieces transposed in frequency. The 10 test trials played by piano were removed and two high and two low frequency trials added to each of five subtests (20 additional). The modified cMBEA was completed by 14 children using bilateral cochlear implants and 23 peers with normal hearing. Results were compared with performance on the original version of the cMBEA previously reported in groups of similar aged children: 2 groups with normal hearing (*n* = 23: Hopyan et al., 2012; *n* = 16: Polonenko et al., 2017), 1 group using bilateral cochlear implants (CIs) (*n* = 26: Polonenko et al., 2017), 1 group using bimodal (hearing aid and CI) devices (*n* = 8: Polonenko et al., 2017), and 1 group using unilateral CI (*n* = 23: Hopyan et al., 2012). Children with normal hearing had high scores on the modified version of the cMBEA and there were no significant score differences from children with normal hearing who completed the original cMBEA. Children with CIs showed no significant improvement in scores on the modified cMBEA compared to peers with CIs who completed the original version of the test. The group with bilateral CIs who completed the modified cMBEA showed a trend toward better abilities to remember music compared to children listening through a unilateral CI but effects were smaller than in previous cohorts of children with bilateral CIs and bimodal devices who completed the original cMBEA. Results confirmed that musical perception changes with the type of instrument and is better for music transposed to higher rather than lower frequencies for children with normal hearing but not for children using bilateral CIs. Overall, the modified version of the cMBEA revealed that modifications to music do not overcome the limitations of the CI to improve music perception for children.

## Introduction

In the present study, we used a modified version of the *child’s Montreal Battery of Evaluation of Amusia* (cMBEA) (Peretz et al., 2003, 2013) to assess music perception in children using bilateral cochlear implants. Our overall aim was to help children with bilateral CIs discriminate fundamental aspects of music. To do this, we modified the complement of instruments used in the cMBEA and added pieces which were shifted in frequency.

### Music in Childhood

Music is considered by many to be a universal language and has been present throughout history in every culture. Musical abilities develop in the early months of life (Trehub, 2001). Infants can detect changes in various aspects of musical stimuli, such as *contour* (pitch direction), *interval* (pitch changes that preserve melodic contour), *scale* (tonality), and rhythm. Despite differences in the acoustic features of music and speech (Smith et al., 2002) and hemispheric specializations for spectral and temporal processing (Zatorre et al., 2002), musical development parallels language development and may be critical to language acquisition in humans (Brandt et al., 2012; Norman-Haignere et al., 2019). In fact, musical training increases cortical plasticity, which can strengthen common subcortical circuits and lead to widespread benefits in diverse non-musical skills, such as speech perception in noise, auditory attention, and auditory working memory (Jäncke, 2012; Kraus et al., 2012).

Music perception is a difficult task, which recruits diverse brain regions (Limb et al., 2010). The melodic (pitch-based *what*) and temporal (time-based *when*) dimensions of music are analyzed in parallel by separate neural subsystems (Peretz et al., 2003). The auditory cortex plays a major role in the processing of pitch relations, while temporal relations are also computed by distinct regions, such as the motor cortex, cerebellum, and basal ganglia (Peretz and Zatorre, 2005). Specifically, the right auditory cortex is specialized for pitch processing, whereas the left auditory cortex plays a more important role in rhythm perception (Zatorre et al., 2002).

### Music Perception in Cochlear Implant Users

Music perception in listeners with normal hearing is facilitated by the presence of low-order resolved harmonics (Plomp, 1967), which are not well-represented by CI devices with poor frequency and temporal fine structure resolution, due in part to few electrodes, current spread, envelope-based processing, and low stimulation rates (e.g., Zeng, 2002). CIs provide fewer than eight effective channels, but music and pitch perception continue to improve when channels increase (up to ∼60) in normal listeners (Kong et al., 2004; Mehta and Oxenham, 2017). Although current spread prevents distortion of binaural processing by small place mismatches, it also reduces the number of independent channels represented by the CI, thereby limiting the capacity for pitch discrimination and music perception (Jiam and Limb, 2019). New speech processing strategies attempt to provide better fine temporal resolution but with little effect on music perception (Magnusson, 2011).

Given effects of CI processing on music, it is not surprising that adults receiving cochlear implants rated their music appreciation and enjoyment as decreasing from a mean of 8.7/10 before hearing loss to 2.6/10 after implantation (Leal et al., 2003; Mirza et al., 2003). The first French patient to receive electrical stimulation from within the cochlea famously described the changes in stimulation from different cochlear places (in order to stimulate changes in pitch perception) as “the turning of a roulette wheel” (Djourno and Eyries, 1957; Djourno et al., 1957). Studies since then have shown that CI users tend to perceive rhythm in music more accurately than pitch or timbre (McDermott and McKay, 1997; Gfeller et al., 2002b; McDermott, 2004; Bruns et al., 2016). The minimum interval that CI users can discriminate is larger than seven semitones on average, compared with well under 1 semitone for NH peers (Gfeller et al., 2002a; Mary Zarate et al., 2012; Bruns et al., 2016). When presented with pairs of sound sequences varying in rhythm, but not pitch, and asked to determine whether they are the same or different, adult CI users achieved a mean score of 88% (Gfeller and Lansing, 1991). Similarly, melodies with more rhythmic patterns were more easily recognized (Schulz and Kerber, 1994). Increased activation in the frontal cortex during melody versus rhythm perception may reflect greater mental effort (Limb et al., 2010).

Children with CIs perform even more poorly on music perception and recognition tests than adult CI users (Jung et al., 2012) but their ratings of musical enjoyment can be high nonetheless (Drennan et al., 2015). Children are better able to hear changes in rhythm than aspects of music which require spectral resolution [e.g., scale, contour, or interval (Hopyan et al., 2012) or harmony (Zimmer et al., 2019)]. Music perception is slightly better in those children who had some residual hearing during the period prior to implantation (Hopyan et al., 2012). Children can successfully combine residual acoustic hearing in one ear with electrical hearing through a CI in the other ear (bimodal hearing) to discriminate differences between music excerpts (Polonenko et al., 2017). Children who have access to hearing in both ears through two CIs or a hearing aid in one ear and CI in the other (bimodal) are able to discriminate some musical changes better than children who use one CI alone (Polonenko et al., 2017). The ability to use mode (i.e., pitch) cues to judge emotion in music increases with longer periods of acoustic hearing prior to implantation and better residual hearing in the non-implanted ear (Hopyan et al., 2012; Giannantonio et al., 2015). Music perception and singing also appear to improve for children with CIs who receive musical training (Giannantonio et al., 2015; Polonenko et al., 2017; Yang et al., 2019). Moreover, there are reports of individual CI users who have good music perception (Maarefvand et al., 2013), suggesting possibilities for improved music listening through CIs.

### The Montreal Battery of Evaluation of Amusia for Testing Music Perception in Cochlear Implant Users

The *Montreal Battery of Evaluation of Amusia* (MBEA) test (Peretz et al., 2003) has proven to be a sensitive, reliable, and valid tool for identifying impaired music perception in individuals with amusia (Vuvan et al., 2018) as well as in adult and child CI users (Cooper et al., 2008; Hopyan et al., 2012; Polonenko et al., 2017; Lima et al., 2018). The child’s MBEA (cMBEA) has slight differences from the adult version (MBEA) (Peretz et al., 2013); the melodies are shorter (∼7 notes rather than ∼10), there are fewer test items (20 rather than 30 in each subtest), the metric test is not included, and 10 different instruments are used to make the test more engaging for children. The cMBEA has five subtests: *Scale, Contour, Interval, Rhythm*, and *Memory*. Half of the trials in the first four subtests contain identical pairs of melodies, while the other trials are melodies that differ by one note. Children indicate whether the pairs are the “same” or “different.” Note differences can be out-of key (Scale subtest), a change in pitch directions (Contour subtest), a change in note interval within the same the key and contour of the melody (Interval subtest), or a change in grouping of note durations (Rhythm subtest). In the fifth and final subtest, single melodies are presented; half were previously presented in the first four subtests and the other half are new melodies. Children indicate whether each melody is “old” or “new.”

The musical excerpts in the cMBEA have fundamental frequencies ranging from 247 to 988 Hz. The perception of these melodies could be compromised in CI users for a number of reasons. First, as discussed above, there are limited numbers of effective cochlear implant frequency channels (Rubinstein, 2004) to represent the spectral components of the musical stimuli with restricted representation of low frequencies. Second, the range of frequencies contained in the music are represented by electrodes that sit in more basal areas of the cochlea than predicted by frequency-position functions (Greenwood, 1990; Stakhovskaya et al., 2007). This can increase the pitch heard by cochlear implant users. Third, there may be decreasing populations of spiral ganglia available for electrical stimulation in more basal than apical areas of the cochlea in deafness (Leake et al., 1992; Nadol, 1997) which could further compromise central representation of the musical pieces in the MBEA. These factors may explain why adults with CIs judge higher frequency music to be distorted or shrill compared to lower frequency music (Gfeller et al., 2002a). The effects of shifting the spectra of music to lower or higher frequencies on music perception through a CI remains to be determined.

There may also be aspects of the MBEA stimuli which restrict music perception in CI users. Cooper et al. (2008) noted that some musical excerpts in the MBEA have fundamental frequencies which fall below the range of frequency channels in most CIs and recommended transposing these melodies up two octaves to maximize place pitch perception. On the other hand, Gfeller et al. (2002b) found that pure tone frequency discrimination in a group of adult CI users was better at 1600 Hz than 3200 Hz which suggests that an opposite approach, transposing music to lower frequencies might have benefits. Cooper et al. (2008) also noted that melody transposition below the lower limit of fundamental frequencies used in the MBEA would help to define the limits of temporal pitch coding in CI users.

The complement of instruments that are used to play the cMBEA stimuli could also have unique effects on children using CIs. In the adult version of the test, all music was played by piano whereas the child version contains 10 instruments (including piano) to help maintain test engagement (Peretz et al., 2013). It is clear that children using CIs have more difficulties than their normal hearing peers distinguishing between different instruments (Roy et al., 2014) but potential effects of different instruments on cMBEA performance in either group of children has not been studied to our knowledge.

Children with normal hearing do show clear musical preferences. For example, there are gender-based biases that can influence what instrument children choose to play (O’Neill and Boultona, 1996) and infants prefer happy music which has large pitch changes (Corbeil et al., 2013). These preferences could be affected by deafness and CI use. Adults with CIs rate the timbre quality as being different between different types of instruments (Gfeller et al., 2002b) and exhibit poor pitch perception for piano tones in particular (Galvin et al., 2008). It is possible that there are different effects of instrument on music perception in children with CIs given child-based musical preferences and the limited access to acoustic musical input in early sensitive periods of development.

In the present study, we examined whether modifications to musical excerpts in the cMBEA could help children with CIs better discriminate music. Our specific hypothesis was that better scores on the cMBEA can be achieved by children using CIs by removing piano excerpts and including music excerpts with modified spectra.

## Materials and Methods

This study was conducted under the approval of the Hospital for Sick Children’s Research Ethics Board which adheres to the Tri-Council Policy Statement: Ethical Conduct for Research Involving Humans.

### Participants

A modified version of the Child’s MBEA test (modified cMBEA) was administered to 37 children: 23 with normal hearing and 14 with bilateral CIs. Demographic details for the CI users are detailed in Table 1. All child CI users were recruited from the Cochlear Implant Program at the Hospital for Sick Children in Toronto and had bilateral severe-to-profound sensorineural hearing loss that occurred in childhood; hearing loss was progressive in three children. Two children (CI22 and CI29) had a period of usable residual hearing prior to implantation (aided or unaided thresholds of ≤ 40 dB HL at any test frequency). Six children (CI2-8) received their first devices at median 1.74 years of age (range = 0.73–4.96) and were provided with second devices after 5.53 years of unilateral CI stimulation (0.9, 11.23) (sequential bilateral implantation), and 8 children (CI15-29) received their implants simultaneously at a median of 3.12 years of age (range 0.79–12.15). Children received different device generations (Nucleus 24CA, 24CS, or 24RE) and speech processors using the advanced combined encoder (ACE) strategy.

**TABLE 1:** Participant demographic information.

Child	Etiology	CI1			CI2		Inter-implant delay (years)	Age at test (years)	Bilateral CI experience (years)
		Age (years)	Ear	Device	Age (years)	Device			
CI2	Unknown	1.21	R	24CA	8.49	24RE	7.29	13.99	5.39
CI3	Connexin26	2.27	L	24CA	5.58	24RE	3.31	12.14	6.48
CI4	Usher	1.12	L	24CS	4.90	24RE	3.77	11.63	6.67
CI5	Usher	0.73	R	24RE	1.62	24RE	0.90	8.91	7.22
CI6	Unknown	4.96	L	24CS	15.40	24RE	10.44	17.95	2.50
CI8	Unknown	2.92	R	24RE	14.15	24RE	11.23	17.97	3.76
CI15	Connexin26	1.73	Bilateral	24RE	1.73	24RE	0	8.21	6.38
CI18	Unknown	1.28	Bilateral	24RE	1.28	24RE	0	8.20	6.82
CI20	Unknown	4.54	Bilateral	24RE	4.54	24RE	0	9.84	5.19
CI22	Ototoxicity	12.15	Bilateral	24RE	12.15	24RE	0	16.97	4.76
CI23	Connexin26	0.79	Bilateral	24RE	0.79	24RE	0	5.95	5.08
CI24	Unknown	4.51	Bilateral	24RE	4.51	24RE	0	8.62	4.04
CI25	Connexin26	0.95	Bilateral	24CA	0.95	24CA	0	9.45	8.40
CI29	Unknown	8.44	Bilateral	24RE	8.44	24RE	0	13.83	5.27

*Demographic data for 14 bilateral cochlear implant (CI) users who completed the modified cMBEA (group: BCI_Steel).*

High resolution computed tomography scans confirmed normal cochlear anatomy in all but two children: child CI2 had a Mondini malformation (incomplete partition type II) and child CI22 had an enlarged left vestibular aqueduct. Four children had GJB2 gene mutations causing deficiencies in Connexin 26 gap junction protein (Propst et al., 2006), while smaller subsets had Usher Syndrome (*n* = 2) and received ototoxic medications at a young age (*n* = 1). The etiology of deafness was unknown in the remaining seven children.

A group of 23 children with normal hearing (NH) also completed the modified cMBEA. They were matched to the bilateral CI group in terms of age [*t*(35) = 0.17, *p* = 0.87]. The NH and CI groups also reported taking music lessons or classes over similar periods of time, although the range was wider in the NH group [*t*(32.47) = 1.35, *p* = 0.19; NH mean = 3.05 ± 3.39 years; CI mean = 1.96 ± 1.47 years]. Results of these children were compared to scores from previously reported groups of children who completed the original (unmodified) version of the cMBEA (Hopyan et al., 2012; Polonenko et al., 2017). As detailed in Table 2, the Polonenko data include children who used bilateral CIs and bimodal devices and the Hopyan data focused on children with bilateral deafness who used a unilateral CI. Both studies included their own control groups of children with normal hearing (NH). The age at testing was remarkably similar across all groups. Age at implantation was similar in children receiving bilateral CIs (BCI) and they had similar inter-implant periods. Children using unilateral CIs (UCI) received their implants at slightly older ages than the children receiving bilateral CIs (UCI_Hopyan: 5.0 ± 2.9 years, BCI_Steel: 3.4 ± 3.3 and BCI_Polonenko: 1.7 ± 1.2 years), reflecting CI candidacy at this earlier period of the Toronto SickKids implant program. Children using bimodal devices (BM) were also slightly older at implantation (BM_Polonenko: 7.3 ± 4.4 years) given their access to sound through hearing aids in the non-implanted ear. Duration of time-in-sound was calculated as the sum of the duration of CI experience and pre-implant residual hearing in children using CIs and as age for children with normal hearing. Children with CIs had slightly less time in sound than their normal hearing peers.

**TABLE 2:** Demographics of children who completed the modified and unmodified child versions of the MBEA.

	Group	Study	Child MBEA	Devices	*N*	Age at test (years)	Age at CI-1 (years)	Inter-implant delay (years)	Time in sound (years)
CI Users	BCI_Steel	Steel	Modified	Bilateral	14	11.7 ± 3.9	3.4 ± 3.3	2.6 ± 4.1	8.9 ± 3.0
	BCI_Polonenko	Polonenko	Original	Bilateral	26	10.5 ± 1.7	1.7 ± 1.2	2.5 ± 2.8	8.8 ± 1.9
	BM_Polonenko	Polonenko	Original	Bimodal	8	11.0 ± 2.3	7.3 ± 4.4	n/a	9.2 ± 1.8
	UCI_Hopyan	Hopyan	Original	Unilateral	23	12.5 ± 3.9	5.0 ± 2.9	n/a	9.1 ± 3.9
Normal hearing	NH_Steel	Steel	Modified		23	11.9 ± 3.2	n/a	n/a	11.9 ± 3.2
	NH_Polonenko	Polonenko	Original		16	11.8 ± 3.0	n/a	n/a	11.8 ± 3.0
	NH_Hopyan	Hopyan	Original		23	11.7 ± 2.9	n/a	n/a	11.7 ± 2.9

*Unilateral cochlear implant (UCI), bilateral cochlear implant (BCI), bimodal (BM), normal hearing (NH).*

### The Modified Child’s Montreal Battery of Evaluation of Amusia

A modified version of the cMBEA (Lebrun et al., 2012) was created to evaluate music perception. The cMBEA consists of five subtests: *Scale, Contour, Interval, Rhythm*, and *Memory* (detailed in the Introduction), with fundamental frequencies ranging from 247 to 988 Hz. The 10 test trials, which were composed of piano tones in the original child’s MBEA, were removed, because CI users exhibit poor pitch perception for piano tones (Galvin et al., 2008) and 20 additional trials were added to test effects of frequency shifts. High frequency transpositions aimed to give better access to fundamental frequencies of MBEA musical excerpts and to better mimic cochlear frequency-position (Greenwood, 1990; Stakhovskaya et al., 2007) whereas transposition to lower frequencies might alleviate adverse responses to high pitches in music (Gfeller et al., 2002a), take advantage of and better pure tone frequency discrimination at lower than higher frequencies (Gfeller et al., 2002b), and help to define the limits of temporal pitch coding in implant users (Cooper et al., 2008). Two high frequency trials and two low frequency trials were added to each of the five subtests, resulting in 22 trials in each subtest (110 trials). Four trials in each subtest (20 total) were randomly selected for frequency modification. Sample Manager v3.4.1 (Audiofile Engineering, 2011) was used to raise the fundamental frequency of 10 of these trials by 2 octaves and lower the fundamental frequency of 10 trials by 1 octave. Musical stimuli, ranging from 60 to 70 dB SPL, were presented in a 2.13 m × 2.13 m sound-attenuating booth and played through Windows Media Player on a Dell Vostro 1520 laptop computer and external Centrios speaker system (model no. 1410106) at zero degrees azimuth. Levels were calibrated in dBA using a sound-level meter (Larson-Davis 800B). Listeners were seated 1 m away from the speakers.

Following modifications, the modified cMBEA was comprised of 110 test trials and 10 practice trials and lasted approximately 35 min in duration. Each subtest contained 22 test trials. Subtests were applied in the following order: Scale, Counter, Interval, Rhythm, and Memory. Practice trials preceded each subtest and contained stimuli from the original cMBEA which were representative of each subtest. For the first four subtests, half of the trials contained identical pairs of melodies, while the other half consisted of melodies that differed by one note. Children indicated whether the pairs of melodies that they heard were the same or different by pressing one of two buttons. Half of the fifth and final subtest the surprise/incidental memory test, contained melodies previously presented in the first four subtests, while the other half consisted of new melodies. For this subtest, children were asked whether they had heard the melody presented in the preceding subtests or if it was novel. The number of correct discriminations were summed, creating a “correct discrimination” score. These scores are presented as a percentage of total trials.

### Data Analyses

Mixed model regressions were conducted on the correct discrimination scores using the lme4 (Bates et al., 2015) and lmerTest (Kuznetsova et al., 2017) packages in R and Rstudio (Version 1.0.153) (R Core Team, 2018). ANOVAs and pairwise *post hoc* analyses were implemented using the Satterthwaite method to estimate denominator degrees of freedom for *t*-statistics of the mixed models. Significance was defined at *p* < 0.05. Figures were created using the ggplot2 package (Wickham, 2016).

## Results

**FIGURE 1:**
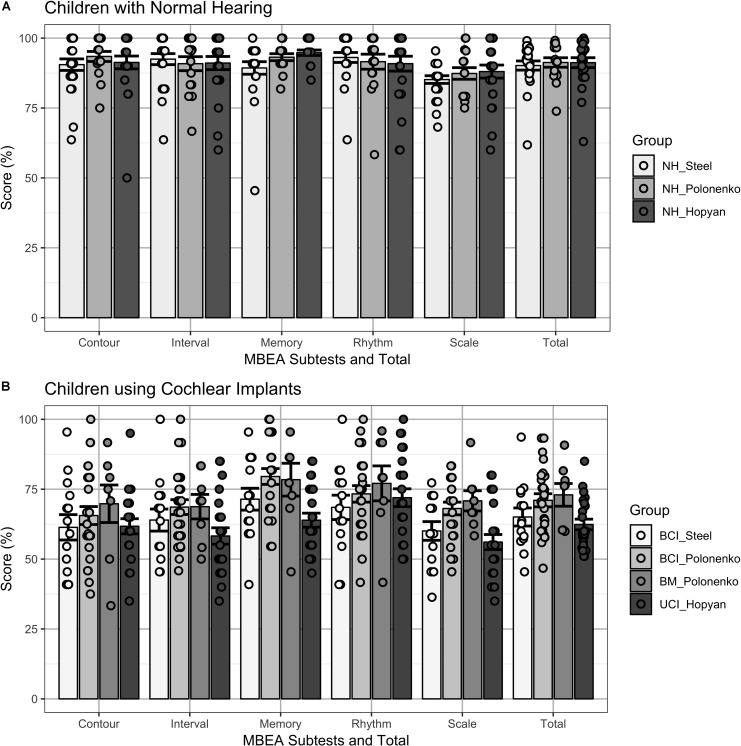
**(A)** Individual (dots) and mean ± SE (bar) scores on cMBEA subtests and the total Score (Total) are shown for three groups of children with normal hearing: children completing the modified cMBEA (NH_Steel), and children who completed the original version (NH_Polonenko and NH_Hopyan). All three groups scored more poorly on the Scale subtest than the other subtests (*p* < 0.05) but there were no significant differences between groups on any of the five subtests or the total score (*p* > 0.05). **(B)** Individual (dots) and mean ± SE (bar) scores on cMBEA subtests and the total score are shown for four groups of children who use cochlear implants. Children with bilateral CI who completed the modified cMBEA (BCI_Steel) and children with Bilateral CIs (BCI_Polonenko), with Bimodal devices (BM_Polonenko) and Unilateral CIs (UCI_Hopyan) who completed the original version of the test from previous studies. Scores were lower than in the children with normal hearing (*p* < 0.0001) with best scores in the rhythm subtests in all groups (*p* < 0.001). In addition, better memory scores were found in the BCI_Polonenko and BM_Polonenko groups than the UCI_Hopyan group (*p* < 0.001 and *p* = 0.004, respectively) and a trend for better memory for the BCI_Steel group relative to the UCI_Hopyan group was found (*p* = 0.07).

Figure 1 plots the individual (dots) and mean ± SE (bar) scores for the groups of children who completed the modified cMBEA (Steel groups) compared to previously published results of children who completed the original version of the cMBEA (Hopyan et al., 2012; Polonenko et al., 2017). Mixed model regression analyses with fixed effects of subtest and group and random intercept for participant revealed significant effects of subtest [*F*(4,504) = 8.5, *p* < 0.0001], group [*F*(6,126) = 34.7, *p* < 0.0001] and an interaction between subtest and group [*F*(24,504) = 2.6, *p* < 0.0001]. Relevant statistical comparisons of the interaction effect are shown in Table 3. The scores of children with normal hearing across studies are shown in Figure 1A.

**TABLE 3:** Significant comparisons of subtest:group interaction.

Group		Subtest comparisons		df	*t*-Value	*p*-Value	Significance
**Subtest comparisons by group**							
NH_Steel	Scale		Contour	504	–0.42	0.034	^∗^
	Scale		Interval	504	–2.39	0.004	^∗∗^
	Scale		Rhythm	504	–2.98	0.002	^∗∗^
NH_Polonenko	Scale		Contour	504	–0.18	0.043	^∗^
NH_Hopyan	Scale		Memory	504	–1.82	0.007	^∗∗^
BCI_Steel	Scale	-	Rhythm	504	–2.14	0.009	^∗∗^
	Scale	-	Memory	504	–5.06	<0.001	^∗∗∗^
	Contour	-	Rhythm	504	–0.84	0.026	^∗^
	Contour	-	Memory	504	–3.76	0.002	^∗∗^
BCI_Polonenko	Scale	-	Rhythm	504	–0.60	0.027	^∗^
	Scale	-	Memory	504	–6.82	<0.001	^∗∗∗^
	Contour	-	Rhythm	504	–3.12	0.001	^∗∗^
	Interval	-	Rhythm	504	–0.09	0.046	^∗^
	Rhythm	-	Memory	504	–1.59	0.008	^∗∗^
BM_Polonenko	Scale	-	Memory	504	0.77	0.075	.
UCI_Hopyan	Scale	-	Rhythm	504	–10.95	<0.001	^∗∗∗^
	Scale	-	Memory	504	–2.90	0.002	^∗∗^
	Contour	-	Rhythm	504	–5.30	<0.001	^∗∗∗^
	Interval	-	Rhythm	504	–8.77	<0.001	^∗∗∗^
	Interval	-	Memory	504	–0.73	0.024	^∗^
	Rhythm	-	Memory	504	12.96	0.001	^∗∗^

**Group comparisons**		**Subtest**		**df**	***t*-Value**	***p*-Value**	**Significance**

**Subtest comparisons across implant groups**							
BCI_Steel vs. other CI groups	Memory	-	Memory:BCI_Polonenko	300	–0.076	0.048	^∗^
	Memory	-	Memory:UCI_Hopyan	300	15.74	0.073	.
BCI_Polonenko vs. other groups	Scale	-	Scale:UCI_Hopyan	300	18.95	<0.001	^∗∗∗^
	Interval	-	Interval:UCI_Hopyan	300	17.29	0.004	^∗∗^
	Memory	-	Memory:UCI_Hopyan	300	22.57	<0.001	^∗∗∗^
BM_Polonenko vs. other groups							
	Scale	-	Scale:UCI_Hopyan	300	24.70	0.004	^∗∗^
	Scale	-	Scale:UCI_Hopyan	300	24.70	0.004	^∗∗^
	Memory	-	Memory:UCI_Hopyan	300	24.45	0.004	^∗∗^

*Significance codes: ‘^∗∗∗^’ < 0.001, ‘^∗∗^’ < 0.01, ‘^∗^’ < 0.05.*

There was no significant difference between the scores of children with normal hearing who completed the modified versus original versions of the cMBEA (NH_Steel vs. NH_Polonenko: *t*(126) = −0.37, *p* > 0.05; NH_Steel vs. NH_Hopyan: *t*(126) = −0.38, *p* > 0.05). Findings in all three normal hearing groups were consistent: all had significantly poorer scores on the Scale subtest compared to at least one other subtest (*p* < 0.05 as detailed in Table 3) and there were no significant differences between the three different NH groups for any of the five subtests or the total scores (*p* > 0.05). Thus, there was no significant effects of the modified version of the cMBEA for children with normal hearing.

Results from the four groups of children using CIs are shown in Figure 1B. As expected, all groups showed significantly poorer scores relative to all three groups of children with normal hearing across subtests and total score (*t* > 4.3, *p* < 0.00001). As previously reported, children with unilateral CIs (Hopyan et al., 2012) and children with bilateral CIs (Polonenko et al., 2017) perceived changes in the Rhythm subtest better than other subtests. This was also true of children with bilateral CIs who completed the modified version of the cMBEA (Steel group). Scores on the memory subtest were also of note: as previously reported, children with bilateral CIs and bimodal users (Polonenko et al., 2017) were able to recall music on the memory subtest better than children with unilateral CIs (Hopyan et al., 2012). Children with bilateral CIs who completed the modified version of the cMBEA showed a similar strength in the memory subtest but the trend toward improvement relative to the Hopyan unilateral CI group did not reach statistical significance (*p* = 0.07).

**FIGURE 2:**
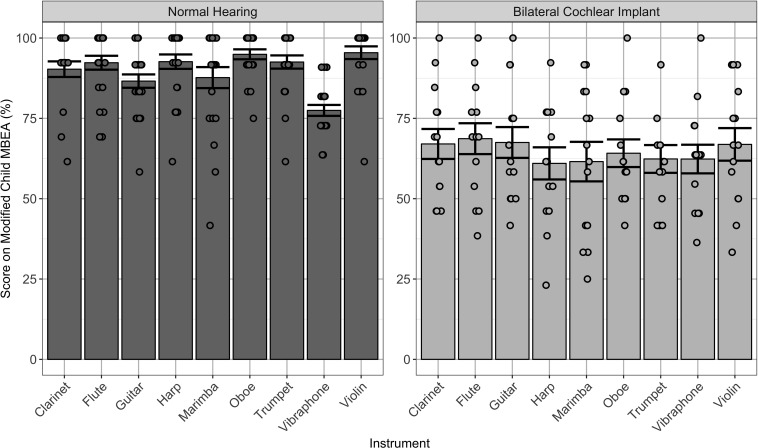
Individual (dots) and mean ± SE (bar) scores on the modified cMBEA stimuli grouped by instrument type. Data from children with normal hearing (NH_Steel), shown on the left, reveal slightly better scores when music was played by the violin (*p* < 0.05). Scores were significantly poorer in children with bilateral CIs (BCI_Steel), shown on the right (*p* < 0.0001), and no significant differences in scores by instrument were found for this group (*p* > 0.05).

As shown in Figure 2, scores across subtests on the modified cMBEA were averaged for music excerpts played by each type of instrument in children with normal hearing and children using bilateral CIs. Significant effects were found for instrument [*F*(8,280) = 3.6, *p* < 0.0001], group [*F*(1,35) = 60.8, *p* < 0.0001] and the interaction between instrument and group [*F*(1,8) = 280, *p* < 0.02]. Comparisons revealed scores in the NH group which were best for the violin and worst for the vibraphone (*p* < 0.05). Scores in children with bilateral CIs were clearly poorer than in their peers with normal hearing (*p* < 0.0001) and were not significantly different between instruments (*p* > 0.05).

**FIGURE 3:**
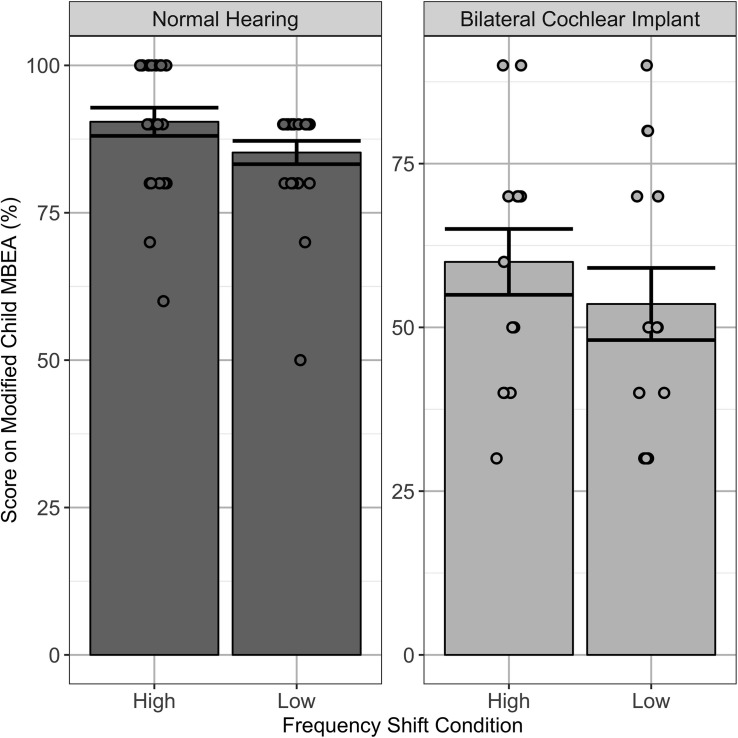
Individual (dots) and mean ± SE (bar) scores on those stimuli in the modified cMBEA which were raised or lowered in frequency. Children with normal hearing (NH_Steel) scored better for music raised to higher frequencies than music lowered in frequency (*p* < 0.05). There was no significant effect for children using bilateral CIs (BCI_Steel) (*p* > 0.05).

Scores on the musical excerpts that were shifted in frequency are shown for both children with normal hearing and children using bilateral CIs in Figure 3. Consistent with overall findings discussed above, scores were significantly poorer in children using bilateral CIs relative to normal hearing peers [*F*(1,35) = 59.0, *p* < 0.0001]. There was also a trend toward differences between high and low frequency shifts [*F*(1,35) = 4.1, *p* = 0.05] which reflected significantly better scores for music shifted to high versus low frequencies (mean ± SD = 90.43 ± 11.47 and 85.22 ± 9.47%, respectively) in children with normal hearing [*t*(22) = 2.4, *p* < 0.05]. Although the mean data suggest the same trends in children with bilateral CI and there was no significant interaction between group and frequency shift [*F*(1,35) = 0.044, *p* = 0.83], the differences in scores between high and low frequency shifts (mean ± SD = 60.00 ± 18.81 and 53.57 ± 20.61%, respectively) were not significant in the CI group [*t*(13) = 1.0, *p* > 0.05].

## Discussion

Results indicated that our modifications to musical excerpts in the cMBEA did not help children with CIs achieve better scores on this test of music discrimination. Specifically, performance by children using CIs on the cMBEA when piano excerpts were removed and music excerpts with modified spectra were included was not different from results in other groups who completed the original version of the cMBEA. This finding was counter to the study hypothesis. The group with bilateral CIs who completed the modified cMBEA showed a trend toward improved abilities to remember music compared to a group listening through a unilateral CI but effects were smaller than in previous cohorts of children with bilateral CIs and bimodal devices who completed the original version of the cMBEA. On the other hand, children with normal hearing did show better music perception for some instruments than others and better scores for music shifted to higher frequencies than music shifted to lower frequencies. Overall, the modified version of the cMBEA revealed that modifications to music do not overcome the limitations of the CI for music perception in children. In addition, trends in the present cohort compliment significant findings in previous groups of children using bilateral devices that show that access to hearing in both ears promotes better memory for music compared to children using unilateral CIs.

### Modifications to the Child MBEA Do Not Affect Discrimination Scores in Children With Normal Hearing

In the modified cMBEA, piano excerpts were removed and 20 trials of musical excerpts which were either raised or lowered in frequency were added. As shown in Figure 1A, these modifications did not affect subtest and total scores in children with normal hearing relative to previous data in similar aged groups who completed the original version of the test. The high scores achieved in all three groups suggest that the distinctions in music tested in the cMBEA are fairly easy for children with normal hearing. Similarly, Vuvan et al. (2018) found that 175 participants aged 16 to 69 years (mean = 29.7 years) with no reported deficits often achieved maximum scores on this test (Vuvan et al., 2018). A slight decrease in score was only found in the Scale subtest and, again, this was a consistent finding across the three groups, confirming that the modified version of the cMBEA had little effect on abilities to detect musical differences in children with normal hearing. The finding that performance was poorer on the Scale subtest may reflect either differences in the discrimination required between the Scale and other subtests or the fact that perception of scale or *tonality* is a more complex and higher-order task than contour and interval perception. The latter point is supported by evidence that musical scale is processed by a specialized system in the prefrontal cortex (Peretz et al., 2003).

### The Modified cMBEA Does Not Yield Better Music Perception Scores in Children Using CIs

Data shown in Figure 1B reveal that the modified cMBEA yields scores that are consistent with those obtained in children with bilateral CIs who completed the original cMBEA in previous studies. Clearly, discrimination scores are reduced relative to normal hearing peers in all 4 of the CI groups. As discussed in previous papers, many children using CIs are effectively amusic (Hopyan et al., 2012; Polonenko et al., 2017; Lima et al., 2018) based on score cutoffs of ∼75% (Vuvan et al., 2018). Yet, unlike individuals with amusia, individuals with hearing loss who use CIs report frequent engagement with music and that they enjoy listening to music (Mirza et al., 2003; Migirov et al., 2009; Looi et al., 2012). This positive relationship with music could stem from a combination of access to music through their CIs, exposure in social and cultural events, and training (Prevoteau et al., 2018; Riley et al., 2018).

Musical experience in children with early onset hearing loss is likely very different from that of adults who lose their hearing and receive implants later in life. Children with CIs hear music in a unique way. As shown in Figure 1B, all of the groups of children with hearing loss were better able to hear changes in the Rhythm subtest as compared with changes on the Scale, Tnterval or Contour subtests. This relative strength was not affected by the modifications to the cMBEA and is consistent with several previous investigations (e.g., Gfeller and Lansing, 1991; Cooper et al., 2008; Hopyan et al., 2012), reflecting adequate temporal resolution through CIs for detecting rhythmic patterns in music (McDermott, 2004). CI users are heavily dependent on rhythm when attempting to identify different melodies (Gfeller et al., 2002a) and struggle to recognize melodies in the absence of rhythm cues (Kong et al., 2004). Rhythm perception may also underlie perception of speech and emotions by CI users (Leal et al., 2003; Hopyan et al., 2011). As Hopyan et al. (2012) noted, music in the Rhythm subtest also contain pitch variations, potentially explaining why children using CIs show poorer scores than normal hearing children on this subtest of the cMBEA. In addition, there may be variability in temporal processing between CI users. Lower gap detection thresholds, one of many possible measure of temporal processing, have been associated with better speech perception in CI users (Muchnik et al., 1994) and perhaps could also predict differences in perception of rhythm in music. Overall, however, the modifications to the cMBEA were either too subtle or targeted the wrong aspects of music to identify relative strengths that children using CIs might have for perceiving music.

### Advantage of Bilateral Input Over Unilateral CI for Music Memory

As shown in Figure 1B, children using bilateral CIs or bimodal devices did not achieve significantly higher scores than children using unilateral CIs on the Scale, Interval, Contour, or Rhythm subtests of the cMBEA (original and modified versions). While a second CI device enhances many binaural listening abilities, such as spatial unmasking, binaural summation, and sound localization (Litovsky et al., 2012), bilateral implantation does not seem to overcome the CI device limitations that compromise music perception in deaf children. Veekmans et al. (2009) used the Munich Music Questionnaire to assess music enjoyment in post-lingually deafened adults who used both unilateral and bilateral CIs and found that a larger percentage of bilateral CI users reported being able to recognize many elements of music, such as melody and timbre, though the difference was not statistically significant. The authors suggested that bilateral implantation may improve music perception by capturing the better ear and reducing the number of cochlear dead regions across the two ears that are not sufficiently stimulated due to lack of neural integrity. It is possible then that music enjoyment is driven more by sound quality than ability to detect differences between musical excerpts. It is important also to keep in mind that adults with post-lingual deafness, like those in the Veekmans et al. (2009) study, were able to access music normally during early development which provides them advantages for music listening over CI users with pre-lingual deafness (Bruns et al., 2016).

The largest difference between bilateral and unilateral CI users in the present cohorts of children was on the Memory subtest. Scores on the memory subtest were significantly better for the bilateral CI and bimodal users in previous cohorts tested with the original version of the cMBEA compared to unilateral CIs users. The bilateral CI users who completed the modified cMBEA showed a trend toward increased Memory subtest scores related to the scores on the original cMBEA in the unilateral CI group (*p* = 0.07). The relative strength of memory for music by children using bilateral devices may be interpreted as a consequence of reduced listening effort in children who have access to bilateral rather than unilateral hearing (Polonenko et al., 2017). The Memory subtest scores were amongst the highest of all subtests in all groups of children using CIs. Hopyan et al. (2012) have pointed out that superior memory for melodies is a phenomenon unique to CI children given that their adult counterparts do not score better on the Memory subtest compared with other subtests on the MBEA (Cooper et al., 2008). If so, perhaps this skill could be harnessed in therapy to improve music perception in children with CIs.

### Music Perception Is Not Better When Particular Instruments or Spectral Changes Are Presented in Children With CIs

Effects of particular instruments and spectral manipulations of music were assessed in children using bilateral CIs and normal hearing peers using a modified version of the cMBEA. Changes in scores, shown in Figure 2, showed effects in the children with normal hearing but not in children with bilateral CIs. As shown, scores were highest for music played by violin and poorest when the music was played with the vibraphone in children with normal hearing. This could reflect biases and preferences of each participant, prior musical exposure, or different discrimination skills required by each subtest. By contrast, children with CIs showed no changes in score by instrument, consistent with previous findings that the subtle differences in timbre by instrument are not available to them (McDermott, 2004) or that they are not able to make use of strategies used by adult CI users to discriminate timbre (Kong et al., 2004; Macherey and Delpierre, 2013). With this in mind, it is unlikely that children using CIs have particularly poor perception of piano music. As shown in Figure 1, removing the piano pieces did not significantly affect cMBEA scores in children with normal hearing or in children with CIs.

Administering test batteries with more appropriate stimuli for children using CIs, such as wider ranges of stimulus frequencies, may provide a more valid assessment of children’s music discrimination ability. The modified cMBEA scores did achieve this objective as scores were poorer for music shifted to lower than higher frequencies. CI users primarily use differences in the place of stimulation within the cochlea, as opposed to the rate of neural firing, to code pitch and changes in pitch (Moore, 2003; Laneau et al., 2004); thus, one solution might be to transpose MBEA melodies up two octaves to maximize place pitch perception or to transpose to lower frequency below the lower limit of fundamental frequencies used in the MBEA in order to define the limits of temporal pitch coding in CI users. This was done in the present study; data shown in Figure 3 confirm that children with normal hearing were better able to discriminate trials that were raised in frequency compared to those in response to music lowered in frequency. These effects were not found in children using CIs. It is thus likely that the challenges of CI pitch coding are larger than the problem of mismatched place pitch coding in the cochlea.

## Conclusion

There was no overall advantage to modifying the cMBEA in any of the subtests or the total score in children using CIs, suggesting that the main challenges to CI processing of music cannot be solved by playing music with specific instruments or transposing music to try to minimize mismatches in place-pitch representation in the cochlea. Rather, potential strengths in memory for music in children with CIs might be harnessed in therapy to help improve their perception of music. Future studies might also take advantage of within-subject testing to assess changes in music perception with interventions by using shorter tests of music perception such as the abbreviated version of the cMBEA (Peretz et al., 2013).

## Data Availability Statement

The datasets generated for this study are available on request to the corresponding author.

## Ethics Statement

The studies involving human participants were reviewed and approved by the Hospital for Sick Children Research Ethics Board. Written informed consent to participate in this study was provided by the participants’ legal guardian/next of kin.

## Author Contributions

MS, MP, SG, and TH collected and analyzed the data, and reviewed the manuscript. BP reviewed and supported the data collection and analyses, and reviewed the manuscript. KG supervised the data collection, analyzed the data, and wrote the manuscript.

## Conflict of Interest

The authors declare that the research was conducted in the absence of any commercial or financial relationships that could be construed as a potential conflict of interest.
